# Molecular Diagnosis of 5α-Reductase Type II Deficiency in Brazilian Siblings with 46,XY Disorder of Sex Development

**DOI:** 10.3390/ijms12129471

**Published:** 2011-12-19

**Authors:** Flávia Leme de Calais, Fernanda Caroline Soardi, Reginaldo José Petroli, Ana Letícia Gori Lusa, Roberto Benedito de Paiva e Silva, Andréa Trevas Maciel-Guerra, Gil Guerra-Júnior, Maricilda Palandi de Mello

**Affiliations:** 1 Center of Molecular Biology and Genetic Engineering (CBMEG), State University of Campinas (UNICAMP), Avenida Cândido Rondon 400, 13083-875, Campinas, SP, Brazil; E-Mails: flaleme@unicamp.br (F.L.C.); soardi@unicamp.br (F.C.S.); rpetroli@unicamp.br (R.J.P.); lenticia_lusa@yahoo.com.br (A.L.G.L.); 2 Interdisciplinary Group of Studies in Sex Determination and Differentiation (GIEDDS), Faculty of Medical Sciences (FCM), State University of Campinas (UNICAMP), 13083-970 Campinas, SP, Brazil; 3 Center of Studies and Researches in Rehabilitation (CEPRE), Faculty of Medical Sciences (FCM), State University of Campinas (UNICAMP), Rua Tessália Vieira de Camargo, 126, 13083-970, Campinas, SP, Brazil; E-Mail: rdepas@fcm.unicamp.br; 4 Department of Medical Genetics, Faculty of Medical Sciences (FCM), State University of Campinas (UNICAMP), Rua Tessália Vieira de Camargo, 126, 13083-970, Campinas, SP, Brazil; E-Mail: atmg@fcm.unicamp.br; 5 Department of Pediatrics, Faculty of Medical Sciences (FCM), State University of Campinas (UNICAMP), Rua Tessália Vieira de Camargo, 126, 13083-970, Campinas, SP, Brazil; E-Mail: gilguer@fcm.unicamp.br

**Keywords:** SRD5A2 deficiency, *SRD5A2* gene mutations, structural analysis

## Abstract

The steroid 5α-reductase type II enzyme catalyzes the conversion of testosterone (T) to dihydrotestosterone (DHT), and its deficiency leads to undervirilization in 46,XY individuals, due to an impairment of this conversion in genital tissues. Molecular analysis in the steroid 5α-reductase type II gene (*SRD5A2*) was performed in two 46,XY female siblings. *SRD5A2* gene sequencing revealed that the patients were homozygous for p.Gln126Arg missense mutation, which results from the CGA > CAA nucleotide substitution. The molecular result confirmed clinical diagnosis of 46,XY disorder of sex development (DSD) for the older sister and directed the investigation to other family members. Studies on SRD5A2 protein structure showed severe changes at NADPH binding region indicating that structural modeling analysis can be useful to evaluate the deleterious role of a mutation as causing 5α-reductase type II enzyme deficiency.

## 1. Introduction

The conversion of testosterone (T) in 5α-dihydrotestosterone (DHT) mediated by 5α-reductase type II enzyme is an essential process for the normal sexual differentiation of male external genitalia during fetal life [1].

The decrease in the DHT synthesis due to mutations in the 5α-reductase type II gene (*SRD5A2*) results in a disorder of sex development (DSD) in individuals with 46,XY karyotype [2–4]. DHT is the most potent androgen responsible for virilization of the external genitalia in embryonic life as well as for prostate differentiation and virilization at puberty. At birth, 46,XY affected individuals may exhibit genital ambiguity or female genitalia but, generally, normal internal reproductive structures [5–8]. In some cases, affected individuals are reared as females that will present spontaneous virilization at puberty [9].

The *SRD5A2* gene is located at 2p23 and comprises five exons separated by four introns occupying approximately 60 kb of genomic DNA. The coding sequence is translated into a polypeptide of 254 amino acids. The protein presents a testosterone binding domain and also a NADPH cofactor-binding domain in the N-terminal region. Mutations that affect the testosterone binding region are located in either exon 1 or 5, while mutations affecting the NADPH binding are more numerous and generally map within exon 3 and 4 [10,11].

In the present study, we have identified the p.Gln126Arg homozygous mutation in exon 2 of *SRD5A2* gene in two 46,XY affected siblings raised as females. The *SRD5A2* molecular diagnosis was important in this family to ensure an early identification of the enzymatic deficiency in a younger sib and to offer further appropriate medical and psychological support.

## 2. Results and Discussion

After studying and analyzing the *SRD5A2* gene in case 1, the clinical suspicion of 5α-reductase type II deficiency was confirmed. The patient was informed about her diagnosis and about the possibility of gender reassignment. She was asked to bring her 10-year-old sister, who had not been diagnosed yet, to be evaluated.

**Figure 1:**
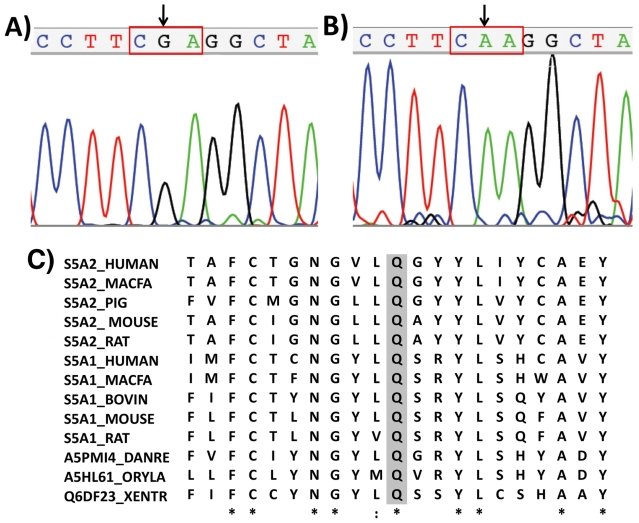
Electropherogram of part of exon 2: (**A**) Homozygous CAA > CGA nucleotide change in codon 126 identified in patient 1; (**B**) Normal CAA sequence in a control individual; (**C**) Multiple amino acid alignments with: human SRD5A2, human SRD5A1 isoenzyme and enzymes of vertebrate animals’ orthologs. The glutamine residue (Q126) is shaded. The UniProt accession numbers for 5α-reductase protein sequences are: *Homo sapiens* (SRD5A2: P31213 and SRD5A1: P18405), *Macaca fascicularis* (SRD5A2: Q28892 and SRD5A1: Q28891), *Sus scrofa* (SRD5A2: O18765), *Mus musculus* (SRD5A2: Q99N99 and SRD5A1: Q68FF9), *Rattus norvegicus* (SRD5A2: P31214 and SRD5A1: P24008), *Bos taurus* (SRD5A1: A5PJS2), *Danio rerio* (SRD5A1: A5PMI4), *Oryzias latipes* (SRD5A1: A5HL61), *Xenopus tropicalis* (SRD5A1: Q6DF23).

All five *SRD5A2* exons were amplified by PCR for both patients. The molecular analysis revealed that both sisters were homozygous for p.Gln126Arg missense mutation. The mutation resulted from the C**A**A > C**G**A nucleotide substitution in the exon 2 (Figure 1). It is considered to completely inactivate the enzyme causing a drastic decrease in its half-life when transfected into mammalian cells *in vitro* [10,12]. This mutation had been previously described in Brazilian, Portuguese, French, Spanish, German, Belgium and North American patients and, in all cases, it was associated to severe phenotype of SRD5A2 deficiency [1,8–10,13–18]. Hackel *et al.* [8] described the compound heterozygosity for this mutation in four Brazilians patients.

Figure 1(C) shows that Q126 residue is conserved in humans for both SRD5A1 and SRD5A2 isoforms and also for proteins of several vertebrate animal orthologs. Therefore it is located in a very conserved region of the protein suggesting that this residue is very important for enzymatic structure and activity.

**Figure 2:**
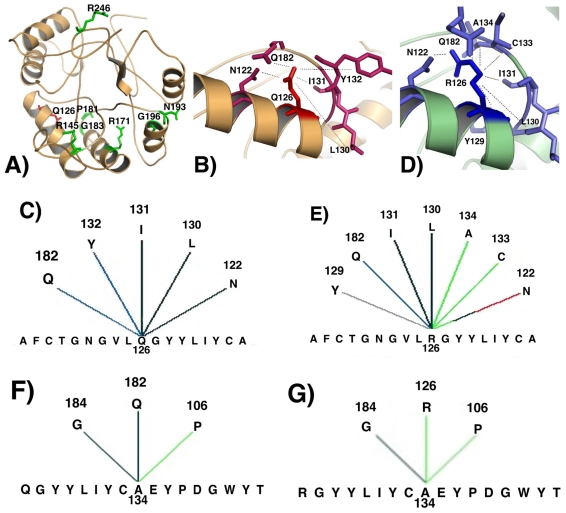

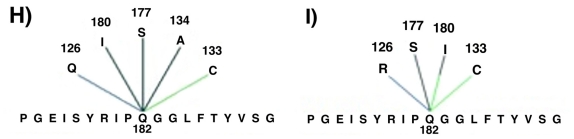
(**A**) Modeled structure for the wild-type human 5α-reductase type II enzyme: Q126 native residue is denoted in red, and NADPH-binding site residues are illustrated in green; (**B**) Part of the modeled SRD5A2 structure showing internal contacts for Q126 native residue; (**C**) Graphical representation for internal contacts of Q126 residue; (**D**) Part of the modeled SRD5A2 structure showing internal contacts for R126 mutant residue; (**E**) Graphical representation for internal contacts of R126 residue: interactions with different residues (A134, C133) are created, one interaction is suppressed (Y132) and two different interactions are established with N122 residue; (**F**) Graphical representation for internal contacts for A134 residue in the native protein: interactions with G184, Q182 and P106 are observed; (**G**) Graphical representation of internal contacts for A134 residue in the mutant protein: the novel interaction with R126 is observed; (**H**) Graphical representation of internal contacts for Q182 residue in the native protein; (**I**) Graphical representation of internal contacts for Q182 residue in the mutant protein: A134 interaction is abolished and a new contact with I180 is observed. Colored lines represent different types of interactions: black = main chain–main chain hydrogen bond; blue = side chain–main chain hydrogen bond; red = side chain–side chain hydrogen bond; gray = aromatic stacking; green = hydrophobic interaction.

The biological importance of p.Gln126Arg change upon the structure of the enzyme was investigated by modeling the mutant enzyme and comparing it to its native form. Because the crystallographic structure of SRD5A2 is not resolved yet we used Blast algorithm to search for a similar structure to be used in modeling analysis [19]. Around 30% similarity is usually required between sequences to obtain a reliable structural model [20], therefore the human liver 5β-reductase (AKR1D1) (PDB ID: 3G1R-chain A) that presented a similarity of 28.9% was chosen, since there was no other crystalographically resolved protein with higher similarity. The AKR1D1 is a human steroid 5β-reductase and belongs to the aldo/keto reductase family [21]. It is involved in the bile acid biosynthesis and also participates in the initial step of steroid hormone metabolism. In humans, steroid 5β-reductases (SRD5A1, SRD5A2) and steroid 5β-reductase (AKR1D1) act to yield the corresponding 5α- or 5β-dihydrosteroids, respectively [1,22]. The modeled structure indicated that p.Gln126Arg is located within an internal region of the protein near the NADPH-binding region (Figure 2(A)). The NADPH-binding domain in SRD5A2 comprises residues R145, R171, P181, G183, N193, G196 and R246 [10]. Observing the structural model, those amino acids are organized as a pocket to receive NADPH (Figure 2(A)). Different interactions were shown for either Q126 native residue or R126 mutant residues when comparing internal contacts (Figures 2(B–E)). Hydrogen bond interactions with Q182 (main chain–side chain) and with both I131 and L130 (main chain–main chain) were maintained in both native and mutant proteins, whereas the aromatic interaction with Y132 was abolished and two different interactions with N122 (one hydrophobic and other hydrogen bond main chain–side chain) were established in the mutant. In addition, the R126 mutant residue demonstrated three different internal interactions: an aromatic stacking with Y129 and two hydrophobic interactions with A134 and C133. Either native or mutant 126 residue in SRD5A2 protein are linked through a hydrogen bond to Q182 residue which is located within the NADPH-binding site region. However, the new hydrogen bond interaction between A134 and R126 mutant residue might affect the structure of NADPH-binding domain by disrupting the native interaction between A134 and Q182 residues (Figures 2(F,G)) and creating novel hydrophobic interaction between Q182 and I180 residues (Figures 2(H,I)).

Those results indicate that p.Gln126Arg mutation probably modify or abolish 5α-reductase type II enzyme activity by preventing NADPH binding.

## 3. Experimental Section

### 3.1. Patients

**Figure 3:**
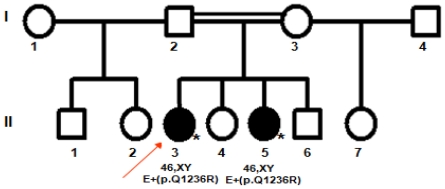
Family pedigree.

A 17-year-old patient (case 1), reared as a girl, was referred to us to investigate virilization by the time of puberty. She was born at term after an uneventful pregnancy and normal delivery. She was the first child of consanguineous parents (first cousins); there were 3 paternal and 2 maternal half sibs, 2 sisters and a brother. According to the patient, an ultrasound of her 10-year-old sister (case 2) revealed absence of uterus, therefore she was also suspected to have sex ambiguity (Figure 3).

On physical examination, her weight was 55 kg and height 175 cm. She exhibited an 8-cm phallus with chordee; a single perineal opening; no vaginal introitus; labioscrotal folds were fused, pigmented and enrugated; and 10 cm^3^ testis were both palpable—the right was in the inguinal region and the left in the labioscrotal fold. Additionally, she had a male distribution of pubic hair, facial hair was absent and there was no breast development. She demonstrated a male gender behavior and considered herself as homosexual.

The patient karyotype was 46,XY. Upon pelvic ultrasound, uterus was absent and prostate was also not detected. Hormonal evaluation showed elevated FSH (16 IU/L; normal male range (NR) 1.5–12.4), slightly elevated LH (8.7 IU/L; NR 1.7–8.6) and normal total testosterone (13.6 ng/mL; NR 8.8–27.0), and free testosterone (5 pg/mL; NR 2.8–8.1) levels, with low dihydrotestosterone level (0.3 ng/mL; NR 0.5–2.9), and high testosterone/dihydrotestosterone ratio (45.3; NR < 10). After 15 months of follow-up with psychological support, the patient requested sex reassignment to male.

The patient’s sister (case 2) was 11 years old when first examined by us. On physical examination, her weight was 32.3 kg and height 143.6 cm. She exhibited a 2.5-cm phallus with *chordee*, a single perineal opening and no vaginal introitus. Labioscrotal folds were not enrugated or pigmented, and her gonads were not palpable; there were no signs of pubertal development. Her karyotype was 46,XY, and hormonal evaluation revealed prepubertal levels of FSH, LH and testosterone.

When she was 12.5 years old, her phallus measured 4 cm and pubic hair was on Tanner stage 2. A few months later, hormonal evaluation revealed pubertal levels of FSH (7.75 IU/L), LH (5.3 IU/L), and total (9.6 ng/mL) and free testosterone (5.85 pg/mL), low dihydrotestosterone level (0.2 ng/mL), and high testosterone/dihydrotestosterone ratio (48.0). The gonads were not seen upon pelvic ultrasound. When she was last seen by us, at 13.5 years, her phallus measured 5 cm and pubic hair was on Tanner stage 3.

As soon as the diagnosis of 5-alpha-reductase type 2 deficiency was confirmed, a careful approach was conducted to explain her condition, which was favored by her knowledge about what was going on with her older sister. Since then, she has been followed by a specialized psychologist who has evaluated her periodically for her understanding and adapting to male gender. At the beginning, she revealed interest in situations which are socially viewed as male activities, such as playing soccer with boys and watching action movies, and using clothes which were not typically female, although, at first, she did not see herself as a male. At the present, she exhibits overtly male behavior.

### 3.2. Methods

Genomic DNA was obtained from peripheral blood by proteinase K/phenol extraction method. *SRD5A2* gene molecular analysis was performed by PCR amplification of the five exons (Table 1). PCR products were directly sequenced using Big Dye^®^ Terminator Cycle Sequencing Kit V3.1 Ready Reaction (ABI PRISM/PE Biosystems). Sequences were obtained in an ABI 3700 Sequencer (ABI PRISM/PE Biosystems) and were compared to the *SRD5A2* normal sequence (ENSEMBL**—**ENSG00000049319) using Chromas (reduced version-free software) and CLC Sequence Viewer v.6.0 (free software).

Theoretical structure of human 5α-reductase type II has been modeled using human liver 5β-reductase (AKR1D1) (PDB ID: 3G1R- chain A) as template. The models have been created and validated by default settings and parameters of the SWISS MODEL web-served program. The modeled protein structure was produced and analyzed by the web-based program BlueStarSTING [23].

## 4. Conclusions

The present molecular investigation collaborated in guiding the diagnosis of deficiency in 5α-reductase type II in a family. The molecular analysis provided additional support for the psychosocial male orientation for both female affected siblings that, in the case of the older sister, had been defined even before the diagnosis. Therefore, the identification *SRD5A2* gene mutations contributed in confirming the diagnosis and provided an early diagnosis of a non-symptomatic affected member in the family. Additionally, the structural analysis of the mutated protein demonstrated to be a useful and inexpensive tool to evaluate the deleterious role of a mutation as a cause of the deficiency of 5α-reductase type II enzyme.

## Figures and Tables

**Table 1:** Primers used for PCR and sequencing of the *SRD5A2* gene.

Exon	Forward Primer	Reverse Primer	Ta* (°C)	Fragment size (pb)
**1**	GCAGCGGCCCACCGGCGAGGAACA	TGGACGCCGGGAGCAGGGCAGT	66	369
**2**	CAGTGAATCCTAACCTTTCCTCCC	TTGTTAGCTGGGAAGTAGGTGGAG	59.5	243
**3**	AAGCACCACAATCTGGACACAT	CTCCAGGGAAGAGTGAGAGTCTG	59.5	203
**4**	CAATGATTGACCTTCCGATTCTTC	GTTTGGAGAAGAAGAAAGCTACGT	63	238
**5**	TCAGCCACTGCTCCATTATATTTA	TTGACAGTTTTCATCCAGCATTGTG	59.5	171

* *T*a = annealing temperatures used in PCRs.
